# The antidepressant effect of Shexiang Baoxin Pills on myocardial infarction rats with depression may be achieved through the inhibition of the NLRP3 inflammasome pathway

**DOI:** 10.1002/brb3.3586

**Published:** 2024-07-05

**Authors:** Yue Wang, Yuwen Chen, Bingqing Li, Yilu Zhou, Jing Guan, Fanke Huang, Jingjing Wu, Yanyan Dong, Peiyuan Sun, Xue Tian, Jindan Cai, Feng Ran, Qiuting Dai, Jianfeng Lv

**Affiliations:** ^1^ Department of Cardiovascular Medicine Affiliated Renhe Hospital of China Three Gorges University Yichang China; ^2^ Institute of Cardiovascular and Cerebrovascular Diseases China Three Gorges University, Yichang, China

**Keywords:** depression, myocardial infarction, NLRP3 inflammasome, pyroptosis, Shexiang Baoxin Pills

## Abstract

**Background:**

Patients with myocardial infarction (MI) frequently experience a heightened incidence of depression, thereby increasing the risk of adverse cardiovascular events. Consequently, early detection and intervention in depressive symptoms among patients with MI are imperative. Shexiang Baoxin Pills (SBP), a Chinese patent medicine employed for the treatment of MI, exhibits diverse mechanisms targeting this condition. Nevertheless, its therapeutic efficacy on postmyocardial infarction depressive symptoms remains unclear. The aim of this study is to investigate the effectiveness and mechanism of SBP in managing depression during acute myocardial infarction (AMI).

**Methods:**

A rat model combining MI and depression was established, and the rats were randomly divided into four groups: the model (MOD) group, SBP group, Fluoxetine (FLX) group, and Sham group. After 28 days of drug intervention, cardiac function was assessed using echocardiography while behavior was evaluated through sucrose preference test (SPT), forced swimming test (FST), and open‐field test (OFT). Additionally, levels of inflammatory factors in serum and hippocampus were measured along with NLRP3 inflammasome‐related protein expression via Western blotting and immunofluorescence.

**Results:**

SBP can enhance cardiac function in rats with AMI and depression, while significantly ameliorating depressive‐like behavior. Compared to the Sham group, levels of IL‐1β, IL‐18, TNF‐α, and other inflammatory factors were markedly elevated in the MOD group. However, expressions of these inflammatory factors were reduced to varying degrees following treatment with SBP or FLX. Analysis of NLRP3 inflammasome‐related proteins in the hippocampus revealed a significant upregulation of IL‐1β, IL‐18, NLRP3, ASC, caspase‐1, and GSDMD in the MOD group; conversely, these measures were significantly attenuated after SBP intervention.

**Conclusion:**

We have observed a significant amelioration in depression‐like behavior upon SBP administration during the treatment of AMI, suggesting that this effect may be attributed to the inhibition of NLRP3‐mediated pyroptosis. (The main findings are summarized in the graphical abstract in the supplementary file.)

## Graphical abstract

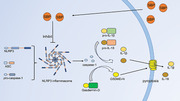

## 1 INTRODUCTION

There is a significant bidirectional association between MI and depression, with approximately two‐thirds of patients hospitalized following a MI exhibiting mild depressive symptoms, while 15% of individuals with cardiovascular disease experience more severe depressive symptoms. Depression is associated with an increased susceptibility to cardiovascular disease, as well as a higher incidence of subsequent mortality and other cardiovascular events (Hare et al., 2014; Jha et al., 2019; Thombs et al., 2006). A prospective cohort study was conducted to investigate the association between Type D personality, depression, and the incidence of major adverse cardiac events (MACE) in patients with AMI. The study enrolled 3568 participants and assessed their baseline levels of Type D personality and depression. Subsequently, a follow‐up period of 2 years was implemented to analyze the occurrence of MACE. The findings from this investigation revealed a higher incidence of MACE among individuals presenting both Type D personality traits and depression (Wang et al., 2023). Furthermore, a meta‐analysis published in 2011 was conducted to investigate the association between depression and mortality rates in patients who had experienced a MI. This comprehensive analysis included 29 studies with a total of 16,889 patients. The data from these studies revealed that individuals with depressive symptoms exhibited a 2.25‐fold increased risk of all‐cause mortality and a 2.71‐fold increased risk of cardiovascular mortality. Moreover, there was an elevated risk of adverse cardiac events by 59% among these patients (Meijer et al., 2011). These findings suggest that the presence of depressive symptoms may contribute significantly to the development of coronary atherosclerosis; thus, it is imperative to assess such symptoms in AMI patients and provide early intervention to mitigate the potential for adverse cardiac events.

Depression is frequently accompanied by the activation of the central inflammatory response, which constitutes a significant factor in depression development (Zhu et al., 2017). The NLRP3 inflammasome plays a pivotal role in this central inflammatory response (Yu et al., 2021). Activation of the NLRP3 inflammasome can initiate caspase‐1‐mediated pyroptosis, resulting in Gasdermin‐D (GSDMD) cleavage by activated caspase‐1. This cleavage liberates the N terminus of GSDMD, leading to pore formation in the cell membrane, cellular rupture, and subsequent cell death. Additionally, it triggers IL‐1β and IL‐18 release, exacerbating inflammation (Liu et al., 2016). Previous studies have demonstrated the presence of NLRP3 inflammasome activation in the hippocampus of depressed rats, which can be effectively suppressed by the antidepressant FLX, resulting in an amelioration of depressive behavior (Fang et al., 2022a). The FLX treatment leads to a reduction in the expression level of IL‐1β in macrophages and microglia, which serves as an inflammatory factor downstream of the NLRP3 inflammasome. This decrease reflects, to some extent, the activity of the NLRP3 inflammasome (Kouba et al., 2022). Moreover, MCC950, an inhibitor of the NLRP3 inflammasome, has been shown to ameliorate depression‐like behavior in mice by suppressing the activation of NLRP3 inflammasome in the hippocampus and reducing the expression levels of NLRP3, ASC, and IL‐1β (Zhai et al., 2018). Notably, animal models of MI (MI) have demonstrated activation of the NLRP3 inflammasome (Zhang et al., 2021; Zhang et al., 2022), suggesting that interventions targeting this pathway may alleviate MI‐associated trauma effectively (Fei et al., 2020; Liang et al., 2020; Zhang et al., 2020). Given the shared involvement of NLRP3 inflammasome in MI and depression pathogenesis, inhibiting its activation could be a promising therapeutic strategy for individuals with comorbid MI and depression.

The SBP, a widely utilized Chinese patent medicine in China renowned for its aromatic and thermogenic properties, comprises a combination of diverse ingredients including artificial musk, ginseng extract, calculus bovis factitius, cinnamon, storax, venenum bufonis, and borneol. These components have traditionally been employed to alleviate symptoms associated with chest paralysis, angina pectoris, MI as well as other conditions caused by qi stagnation and blood stasis. Previous studies have demonstrated the effective inhibition of inflammatory response in mice with MI by SBP resulting in reduced levels of inflammatory factors and subsets of inflammatory monocytes (Lu et al., 2018; Lu et al., 2019). Additionally, one study found that SBP has anxiolytic and antidepressant effects in mice subjected to CUMS (Zhou et al., 2019a). However, it remains unclear whether SBP exhibits similar antidepressant effects in the treatment of AMI and if its mechanism involves inhibiting NLRP3 inflammatory vesicle activation. Therefore, this study aims to investigate the impact of SBP on depression‐like behaviors and the expression levels of NLRP3 inflammatory vesicle‐related proteins in rats with a combined model of AMI and depression, providing a theoretical foundation for identifying therapeutic targets for depression combined with cardiac infarction using SBP.

## 2 MATERIALS AND METHODS

### 2.1 Medicinal materials

The preparation of SBP complies with the Chinese Pharmacopoeia 2015 (Batch numbers: 211125, Shanghai Hutchison Pharmaceuticals, China). SBP contains seven materia medicas, including Artificial Moschus, Calculus Bovis Artifactus, Radix Ginseng, Venenum Bufonis, Cortex Cinnamomi, Styrax, and Borenolum Syntheticum. The major pharmacologically active components are shown in Table 1. The results of qualitative studies on the nonvolatile components of Shexiang Baoxin Pills based on HPLC‐DAD‐ESI‐MS/MS are presented in the Supplementary Material.

**TABLE 1:** The main chemical components of SBP.

Material name	Chinese name	Full scientific name	Major pharmacologically active components
Artificial Mouchus	REN GONG SHE XIANG	The dried preputial secretion of *Moschus berezovskii Flerov, Moschus sifanicus Przewalski*, or *Moschus moschiferus Linnaeus*	Muscone, testosterone
Calculus Bovis Artifactus	REN GONG NIU HUANG	The dried gall‐stone of Bos taurus domesticus Gmelin	Cholic acid, deoxycholic acid, ursodeoxycholic acid, chenodeoxycholic acid, hyodeoxycholic acid, bilirubin, cholesterol
Radix Ginseng	REN SHEN	*Panax ginseng* C.A. Mey., root	Ginsenoside Ra1/2, Rb1/2/3, Re, Rc, Rd, Re, Rf, and Rg1/2/3
Venenum Bufonis	CHAN SU	The dried secretion of *Bufo bufo gargarizans Cantor* or *Bufo melanostictus Schneider*	Cinobufagin, resibufogenin, resibufagin, gamabufotalin, bufalin, 1β‐hydroxylbufalin, arenobufagin, bufotalin, telocinobufagin, telibufagin
Cortex Cinnamomi	ROU GUI	*Cinnamomum cassia* (L.) J. Presl., bark	Cinnamic acid, cinnamaldehyde
Styrax	SU HE XIANG	*Liquidambar orientalis* Mill., resin	Benzyl benzoate
Borneolum Syntheticum	BING PIAN	*Borneolum Syntheticum* or *Dryobalanops aromatica* C.F. Gaertn, resin	Borneol, isoborneol

### 2.2 Experimental animals

**FIGURE 1:**
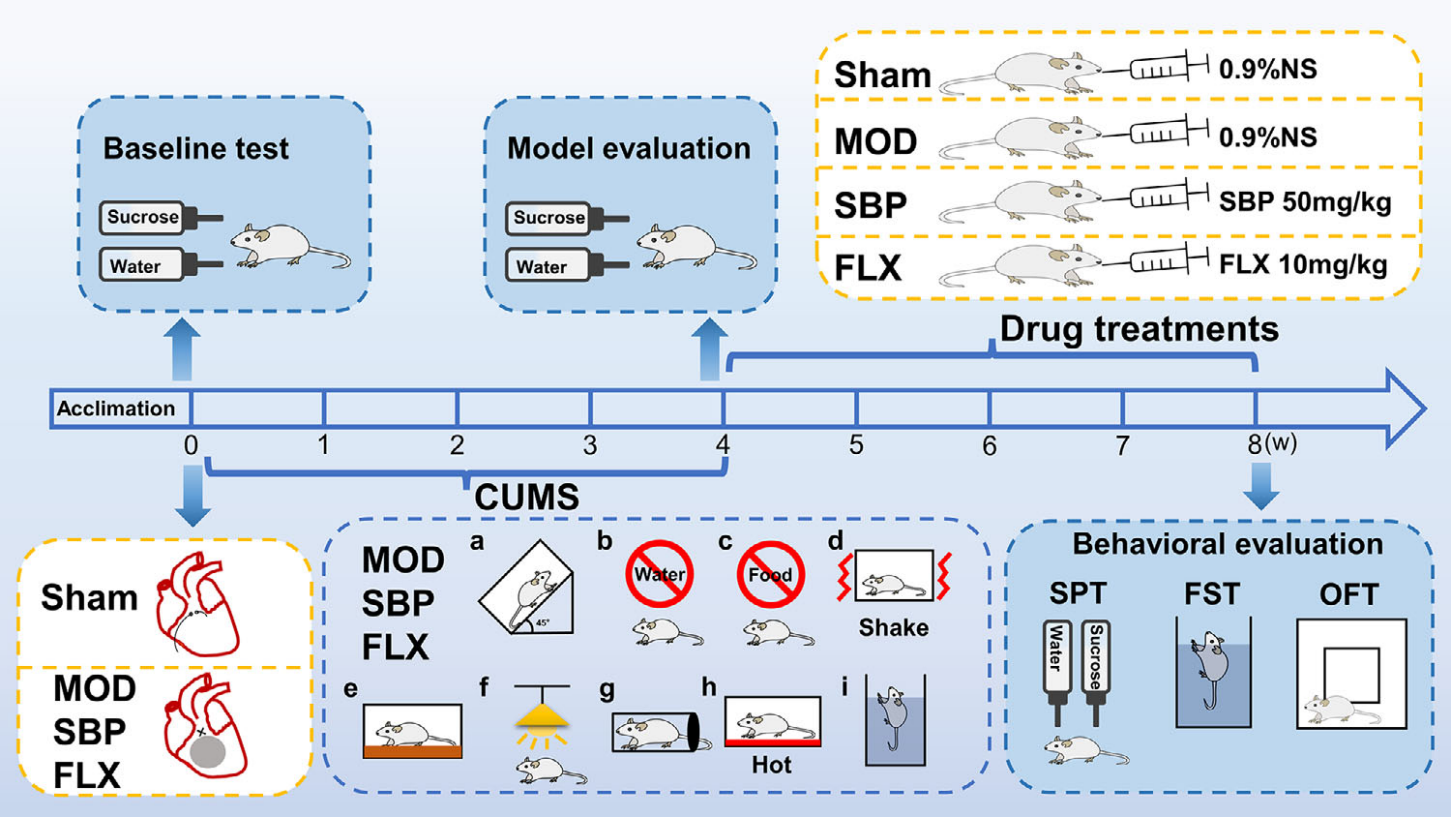
The experimental timeline. Following a 1‐week period of adaptation, baseline behavioral data were collected using the SPT. Subsequently, the LAD ligation surgery and CUMS procedure were conducted on the MOD, SBP, and FLX groups. The depression model was assessed using the SPT at the fourth week. Drug treatments were administered from weeks 4 to 8. At the conclusion of the eighth week, behavioral evaluations were conducted using the SPT, FST, and OFT. CUMS procedure: cage tilting (a); water deprivation (b); food deprivation (c); shaker stress (d); soiled cage (e); continuous overnight illumination (f); physical restraint (g); hot stress (h); forced swimming (i). SPT, sucrose preference test; LAD, left anterior descending coronary artery; CUMS, chronic unpredicted mild stress; MOD, model group; SBP, Shexiang Baoxin Pills group; FLX, fluoxetine group; FST, forced swimming test; OFT, open‐field test.

SPF male Sprague‐Dawley rats weighing 200 ± 20 grams were obtained from the Laboratory Animal Center of China Three Gorges University. The animal experiments conducted in this study adhered to the guidelines for animal care and were approved by the Experimental Animal Ethics Review Committee of China Three Gorges University (2021080N1). Prior to the experiment, the rats were acclimated to their environment for 1 week. Throughout the experiment, the temperature of the experimental environment was maintained at 23–26°C, with a humidity level of 50%−60%. The rats were randomly divided into four groups, each consisting of eight rats: Sham operation group (Sham), Model group (MOD), SBP group, and FLX group. The MI combined with depression model was constructed in the MOD group, SBP group, and FLX group by performing left anterior descending artery (LAD) ligation and chronic unpredictable mild stress (CUMS). The process of creating the acute myocardial infarction model involved anesthetizing rats with sodium pentobarbital, immobilizing them on the operating table, and continuously monitoring their limb lead electrocardiogram. Subsequently, intubation was performed, a small animal ventilator was attached to assist with breathing, and the chest area of the rat was shaved. The surgical site was sterilized, and the surgical field was exposed. Following this, the rat's breathing was facilitated. A 6‐0 black silk thread was punctured approximately 2 mm below the proximal end of the left anterior descending coronary artery, and the vessel was ligated. This resulted in significant ST segment elevation on the limb‐lead electrocardiogram, indicating successful ligation. The physiological and anatomical structure, as well as the negative thoracic pressure of the rats, were restored. At the conclusion of the operation, the ventilator was removed, vital signs were monitored, the tracheal tube was extubated, and the neck muscles and skin were sutured. CUMS procedure: cage tilting; water deprivation; food deprivation; shaker stress; soiled cage; continuous overnight illumination; physical restraint; hot stress; forced swimming. The stressor was given randomly daily for 4 weeks, but the same stressor was not given for 2 consecutive days. In the Sham group, LAD was punctured but not ligated. The construction of the MI model was based on a previous study (Xiong et al., 2022), while the construction of CUMS was based on another study (Xu et al., 2023). Prior to the experiment, the SPT was conducted to establish baseline levels of depression in each group of rats. The success of the depression model was determined by observing a reduction in sucrose preference rate after CUMS. Once successful modeling was achieved, the SBP group received SBP suspension via oral gavage at a dosage of 50 mg/kg/day in a volume of 2 mL, while the FLX group received fluoxetine hydrochloride tablets suspension via oral gavage at a dosage of 10 mg/kg/d in a volume of 2 mL. The Sham group and MOD group were administered the same volume of normal saline via oral gavage daily. After 28 days, behavioral assessment and echocardiography were conducted (Figure 1).

### 2.3 Evaluation of cardiac function

Following the completion of behavioral testing, the rats were administered anesthesia through an intraperitoneal injection of a 1% sodium pentobarbital solution at a dosage of 5 mL/kg. Once anesthetized, the rats were securely positioned on the operating table of an ultrasound instrument, and the chest area was prepared by shaving and applying a probe with a frequency range of 8–13 MHz. Measurements of the left ventricular posterior wall thickness (LVPW d), interventricular septum thickness (IVS d), left ventricular end systolic diameter (LVESD), and left ventricular end diastolic diameter (LVEDD) were obtained by placing the M‐mode sampling line at the papillary muscle. Afterwards, the left ventricular end systolic volume (LVESV), left ventricular end diastolic volume (LVEDV), left ventricular ejection fraction (EF), and left ventricular fractional shortening (FS) were calculated.

### 2.4 Behavioral tests

#### 2.4.1 Sucrose preference test (SPT)

Following a 48‐h period of sucrose acclimatization training, two bottles were introduced into each rat cage: one containing pure water and the other containing a 1% sucrose solution. The water consumption of each rat was then measured after a 2‐h interval in order to determine the sucrose preference rate. Sucrose preference rate = sucrose water consumption/ (pure water consumption + sucrose water consumption) × 100%.

#### 2.4.2 Forced swimming test (FST)

Prior to the commencement of the experiment, tap water was introduced into a transparent drum measuring 20 cm in diameter and 50 cm in height. The drum had a depth of 30 cm and the water temperature was maintained at 25°C. Subsequently, the experimental rats were promptly placed into the water. Simultaneously, the animal behavior acquisition and analysis system, TopscanLite2.00 (Clever Sys Inc. Reston, Virginia, USA), was activated to record the rats' activities in the water for a duration of 5 min. The recorded behaviors included swimming, which was defined as the rat propelling itself upwards using its front paws and moving around the water surface in the pool. Additionally, immobility behavior was observed, which encompassed the rat remaining stationary on the water surface or making slight movements to maintain body balance and prevent submersion. The duration of time spent engaging in resting behavior was subsequently analyzed.

#### 2.4.3 Open‐field test (OFT)

The experimental setup involved placing the rats in a sterile and unobstructed enclosure measuring 75 cm × 75 cm × 40 cm. The rats’ movements were observed and documented for a duration of 5 min using the TopscanLite2.00 analysis system. This system placed a camera directly above the open field, which could track the movement of rats and synchronize the position information to the computer, and then the computer software calculated the moving distance, moving speed and other information. The parameters measured included the overall distance covered by the rats, the distance covered within the central region of the enclosure, and the duration of time spent in the enclosure.

### 2.5 Enzyme‐linked immunosorbent assay (ELISA)

Once the aforementioned tests were finished, anesthesia was induced using a 1% solution of sodium pentobarbital. Blood was then collected from the abdominal aorta and the heart was promptly extracted for additional analysis. The collected blood samples were left at room temperature for a duration of 2 h, after which they were centrifuged at a temperature of 4°C for 10 min at a speed of 3000 revolutions per minute. The upper layer of serum was separated, and the levels of IL‐1β, IL‐18, and TNF‐α in the serum were measured using the ELISA method. IL‐1β, IL‐18, and TNF‐α ELISA kits were purchased from CLOUD‐CLONE CORP (Wuhan, China). Specific procedures were followed according to the instructions provided in the respective kits.

### 2.6 Western blot analysis

Hippocampal tissues were thawed on ice, and an appropriate amount of tissue lysate was added. The lysate was then fully ground, and the resulting supernatant was centrifuged to obtain protein samples. The protein concentration was determined using the BCA method. Sodium dodecyl sulfate‐polyacrylamide gels were prepared, and equal amounts of proteins from each group were loaded onto the gels. Electrophoresis was performed, and the proteins were transferred to a membrane. The membrane was then blocked with 5% skim milk for 1 h. The primary antibody was diluted in an appropriate diluent and incubated with the membrane for 12 h at 4°C. The cells were washed three times with TBST, followed by the addition of the secondary antibody diluent. The cells were incubated at room temperature for 1 h and then washed three times with TBST. Finally, a developer was used to induce color development, and the resulting images were captured using a chemiluminescence apparatus. Primary antibodies used in the experiments: Anti‐IL‐1β (1∶1000, Servicebio, China), anti‐IL‐18 (1∶1000, Proteintech, USA), anti‐GAPDH (1∶2000, Servicebio, China), anti‐NLRP3 (1∶1000, Servicebio, China), anti‐ASC (1∶1000, BIOSS, China), anti‐Caspase‐1 (1∶1000, Servicebio, China), anti‐GSDMD‐N (1∶1000, Immunoway, USA), Anti‐β‐actin (1∶2000, Servicebio, China). The secondary antibodies Goat Anti‐Mouse IgG‐HRP (1∶5000, Servicebio, China) and Goat Anti‐Rabbit IgG‐HRP (1∶5000, Servicebio, China) were used in the experiments.

### 2.7 Immunofluorescence staining

The brains of the rats were rapidly removed under anesthesia, and the hippocampus was dissected, fixed in formaldehyde, dehydrated, embedded in paraffin, and cut into 5 µm thick coronal sections, dewaxed to water, then immersed in EDTA‐Tris solution (pH 9.0) at 98°C for 30 min for antigen repair, and washed 3 times for 5 min with PBS. Subsequently, the slides were incubated with 10% nonimmune goat serum for 30 min at room temperature to block nonspecific staining. After that, the slides were incubated overnight at 4°C with primary antibodies as follows: Anti‐NLRP3 (1:200, Servicebio, China), Anti‐Iba‐1 (1:500, Servicebio, China), Anti‐GSDMD (1:100, Immunoway, USA). Tissues were covered with secondary antibodies labeled with the corresponding species of the primary antibody and incubated for 50 min at room temperature. Finally, the nuclei were counterstained with DAPI. Finally, all stained specimens were observed under a confocal laser scanning microscope. Colocalization analysis was performed by ImageJ software.

### 2.8 Quantitative real‐time RT‐PCR

The hippocampus of rats was dissected and total RNA was extracted using TRIpure Total RNA Extraction Reagent (ELK Biotechnology, USA) following the manufacturer's instructions. Subsequently, cDNA synthesis was performed using EntiLink™ 1st Strand cDNA Synthesis Super Mix (ELK Biotechnology, USA). Quantitative real‐time PCR analysis was conducted with EnTurbo™ SYBR Green PCR SuperMix kit (ELK Biotechnology, USA) and gene‐specific primers. Each sample had three replicate wells. The ΔΔCt method was employed to compare Ct values obtained from different samples. Glyceraldehyde 3‐phosphate dehydrogenase (GAPDH) served as the internal reference gene. The primers used for PCR are listed in Table 2.

**TABLE 2:** Primers used for quantitative real‐time RT‐PCR.

cDNA	sense	antisense
GAPDH	5′‐GCCAAGGTCATCCATGACAAC‐3′	5′‐GTGGATGCAGGGATGATGTTC‐3′
IL‐1β	5′‐GTGGCAGCTACCTATGTCTTGC‐3′	5′‐CCACTTGTTGGCTTATGTTCTGT‐3′
IL‐18	5′‐GCTCTTGTGTCAACTTCAAAGAAAT‐3′	5′‐CAGCCAGTCCTCTTACTTCACTATC‐3′
NLRP3	5′‐AACCAGAGCCTCACTGAACTGG‐3′	5′‐AGAGCAGATGCTTCAGTCCCAC‐3′

### 2.9 Statistical methods

The measurement data were expressed as means and standard deviation (mean ± SD). ANOVA was employed to compare the groups. The gray value of the bands was analyzed using Image J (NIH, USA), while data analysis and mapping were performed using Graph Pad Prism 8.0 (GraphPad Software Inc., San Diego, CA, USA). A significance level of *p* < .05 was deemed statistically significant for the data.

## 3 RESULTS

### 3.1 The administration of SBP effectively attenuated the decline in cardiac function observed in a rat model of MI complicated with depression

**FIGURE 2:**
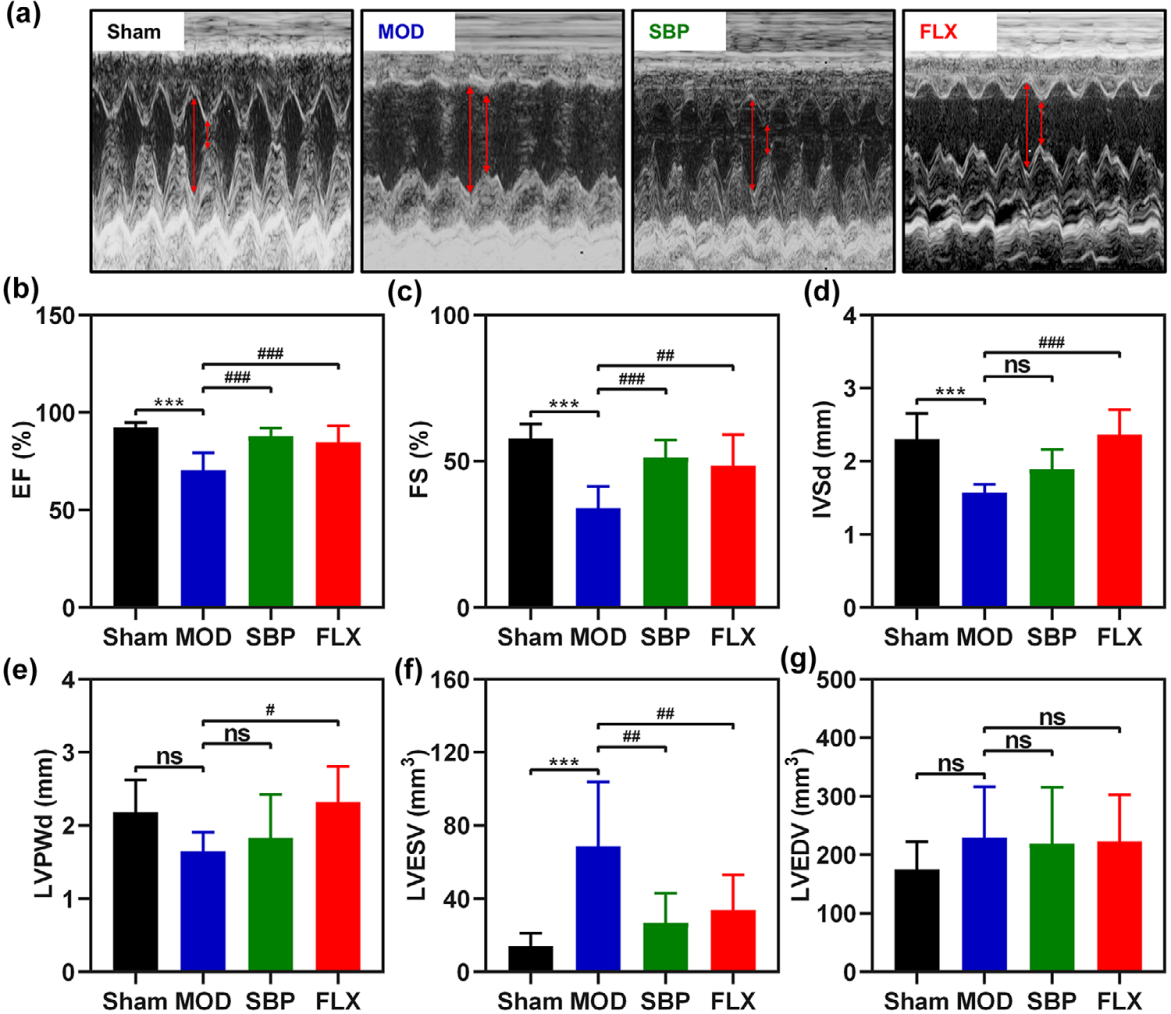
Effects of Shexiang Baoxin Pills on echocardiographic findings in rats with acute myocardial infarction and depression. Representative echocardiographic M‐mode images of rats after 4 weeks of drug intervention (a). Comparison of EF (b), FS (c), IVSd (d), LVPW d (e), LVESV (f), and LVEDV (g) results for each group (*n* = 8). Statistics are presented as mean ± SD and compared by one‐way ANOVA. ^***^ *p *<  .001 vs. Sham group; **^#^** *p *<  .05, **^##^** *p *<  .01, **^###^** *p *<  .001 vs. MOD; ns, not significant. EF, left ventricular ejection fractions; FS, left ventricular fractional shortening; IVS d, interventricular septum thickness; LVPW d, left ventricular posterior wall thickness; LVESV, left ventricle end systolic volume; LVEDV, left ventricular end diastolic volume; MOD, model group; SBP, Shexiang Baoxin Pills group; FLX, fluoxetine group.

To investigate the potential cardioprotective effects of SBP in rats with concurrent MI and depression, echocardiography was employed to evaluate cardiac structure and ejection function in each experimental group. The results demonstrated a significant reduction in cardiac contractility and ejection fraction in rats subjected to ligation of the left anterior descending artery (LAD) and CUMS, which were effectively reversed by SBP treatment. Additionally, fluoxetine hydrochloride, a commonly used medication for alleviating depressive symptoms, also exhibited certain improvements on myocardial contractility and ejection fraction. This is illustrated in Figure 2a where it is evident that the MOD group experienced a significant decrease in ejection fraction while both the SBP group and FLX group showed significant increases.

### 3.2 SBP ameliorated depression‐like behavior in a rat model of MI complicated with depression

**FIGURE 3:**
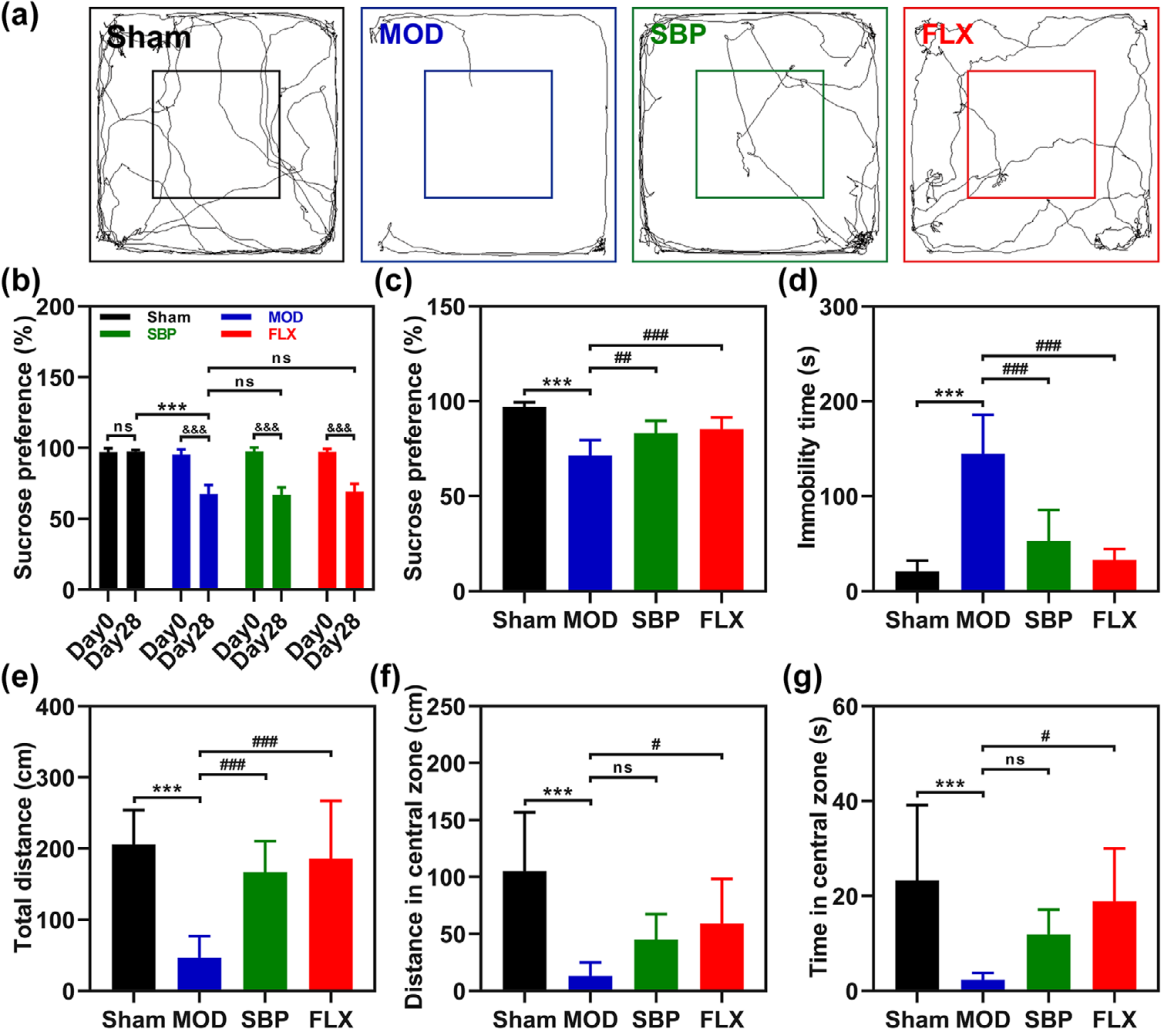
Effects of Shexiang Baoxin Pills on behavioral outcomes in rats with acute myocardial infarction complicated with depression. Representative moving trajectories for each group in open‐field test (a). Comparison of baseline sucrose consumption rate and sucrose consumption rate after CUMS in each group of rats (b). Sucrose consumption rate of rats in each group after 4 weeks of drug intervention in sucrose preference test (c). Immobility time in 5 min for each group of rats in forced swimming test (d). In the open‐field test, the total moving distance (e), central zone moving distance (f), and central zone retention time (g) of rats in each group. Statistics are presented as mean ± SD and compared by one‐way ANOVA with Tukey's post hoc for between groups and independent samples *t*‐test for within‐group (*n* = 8) **^&&&^** *p *<  .001 vs. the same group before CUMS; ^***^ *p *<  .001 vs. Sham group; **^#^** *p *<  .05, **^##^** *p *< .01, **^###^** *p *<  .001 vs. MOD; ns, not significant. CUMS, chronic unpredicted mild stress; MOD, model group; SBP, Shexiang Baoxin Pills group; FLX, fluoxetine group.

To investigate the potential antidepressant effect of SBP, a depression model was established in rats using CUMS. Fluoxetine was employed as a positive control. Prior to and after the implementation of CUMS, baseline depression‐like behavior was assessed using the SPT to confirm successful modeling. The results demonstrated a significant reduction in sucrose preference rate among rats subjected to CUMS for 28 days (Figure 3b). At the fourth week of intervention, behavioral tests were conducted across all groups. The results revealed that the MOD group consistently exhibited significantly lower rates of sucrose preference compared to the Sham group. In contrast, both the SBP and FLX groups demonstrated higher sucrose preference rates in comparison to the MOD group (Figure 3c). This suggests that SBP, similar to fluoxetine, may alleviate anhedonia in rats. The forced swim test (FST) results revealed a significant increase in immobile behavior duration in the MOD group compared to the Sham group, while both the SBP and FLX groups exhibited reduced resting times (Figure 3d). Moreover, the OFT results demonstrated increased total movement trajectory distance for both the SBP and FLX groups following 28 days of drug intervention (Figure 3e), accompanied by elevated distance (Figure 3f) and time (Figure 3g) spent in central areas. Based on these findings, it can be concluded that SBP effectively ameliorates depression‐like behaviors in rat models induced by AMI.

### 3.3 The administration of SBP attenuated the inflammatory response in rats with MI and comorbid depression

**FIGURE 4:**
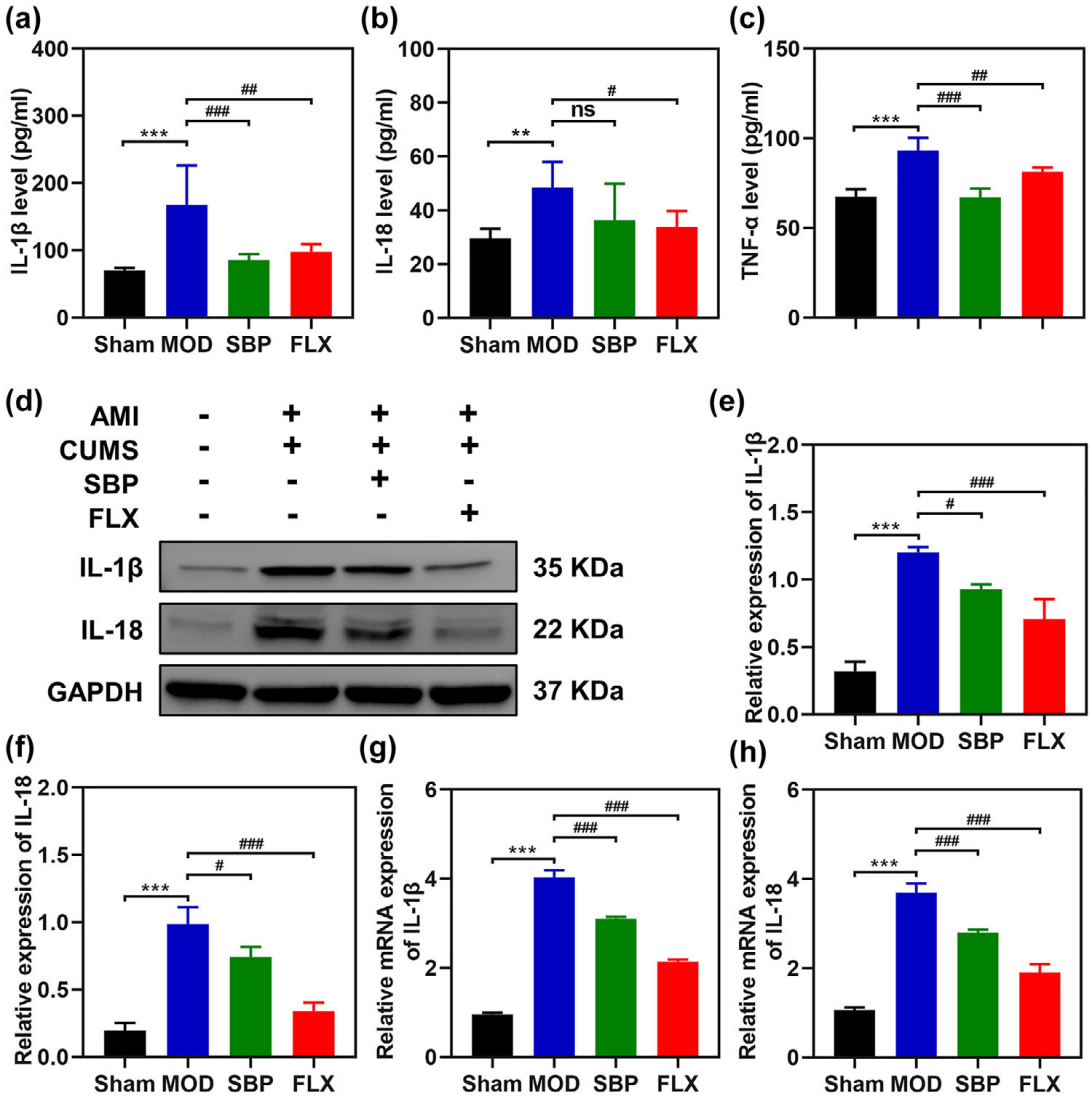
Effects of Shexiang Baoxin Pills on inflammatory factors. The expression levels of IL‐1β (a), IL‐18 (b), and TNF‐α (c) in serum were detected by ELISA. *n* = 6. The Western blotting of IL‐1β and IL‐18 (d, e, and f). *n* = 3. The mRNA expression of IL‐1β (g) and IL‐18 (h). *n* = 3. ^***^ *p *<  .001 vs. Sham group; **^#^** *p *<  .05, **^##^** *p *<  .01, **^###^** *p *<  .001 vs. MOD; ns, not significant. MOD, model group; SBP, Shexiang Baoxin Pills group; FLX, fluoxetine group; AMI, acute myocardial infarction; CUMS, chronic unpredicted mild stress.

Reactive inflammation is observed following AMI, which has been documented to exhibit a significant correlation with depressive symptoms (Beurel et al., 2020). To confirm the presence of inflammation in rats with MI and depression, we quantified the levels of IL‐1β, IL‐18, and TNF‐α in rat serum using ELISA. As illustrated in Figure 4a–c, the serum concentrations of IL‐1β, IL‐18, and TNF‐α were significantly elevated in the MOD group compared to the Sham group (*p* < .05). Conversely, both SBP and FLX groups exhibited a significant reduction in serum levels of IL‐1β and TNF‐α when compared to the MOD group. However, no statistically significant difference was observed for IL‐18 between the MOD group and SBP group (*p *= .074). These findings suggest that MI complicated by depression is associated with increased expression of circulating inflammatory factors; moreover, SBP demonstrate an inhibitory effect on inflammatory response. Previous studies have indicated a link between brain inflammation, particularly within the hippocampus region, and depression (Z. Li et al., 2021). We investigated the mRNA and protein expression of IL‐1β and IL‐18 in the hippocampus. Western blot analysis (Figure 4d–f) revealed an upregulation of inflammatory factors in the MOD group, which was effectively attenuated by treatment with SBP and Fluoxetine. Subsequent qPCR results (Figure 4g and h) further validated these findings, demonstrating elevated mRNA expression levels of IL‐1β and IL‐18 in the MOD group that were significantly reduced following administration of SBP and Fluoxetine. In conclusion, both peripheral blood and hippocampal tissues from rats with MI‐induced depression exhibited heightened levels of inflammatory cytokines that were ameliorated after treatment with SBP and Fluoxetine.

### 3.4 SBP mitigated the activation of microglia and NLRP3 inflammasome in the hippocampus

**FIGURE 5:**
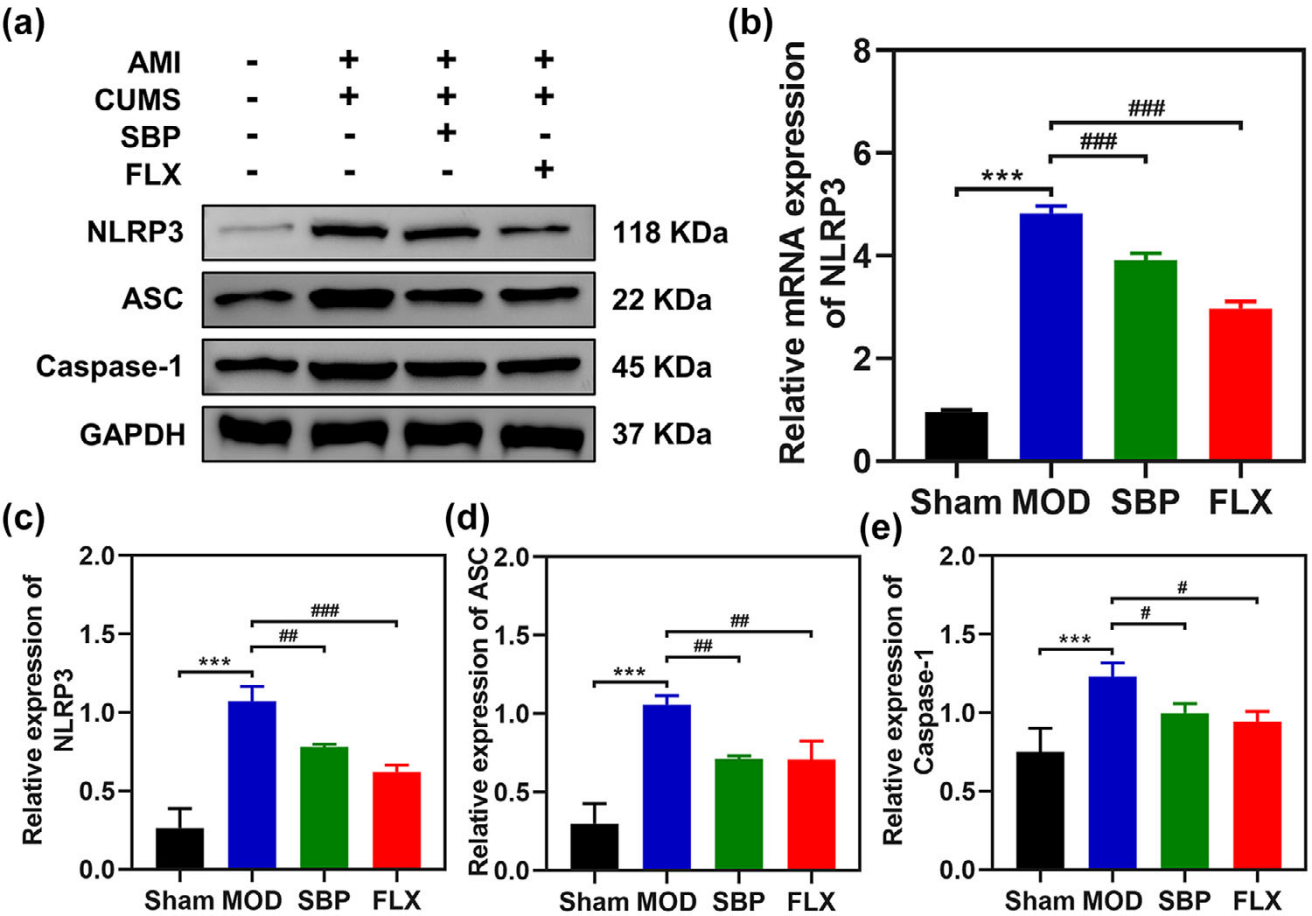
Effect of Shexiang Baoxin Pills on NLRP3 inflammasome in hippocampus. Representative bands detected by Western blotting for NLRP3 inflammasome (a). The mRNA expression of NLRP3 (b). *n* = 3. Analysis of relative gray values of NLRP3 (c), ASC (d), and Caspase‐1 (e) Western blotting bands, as a ratio to GAPDH. *n* = 3. ^***^ *p *<  .001 vs. Sham group; **^#^** *p *<  .05, **^##^** *p *<  .01, **^###^** *p *<  .001 vs. MOD; ns, not significant. MOD, model group; SBP, Shexiang Baoxin Pills group; FLX, fluoxetine group; AMI, acute myocardial infarction; CUMS, chronic unpredicted mild stress.

**FIGURE 6:**
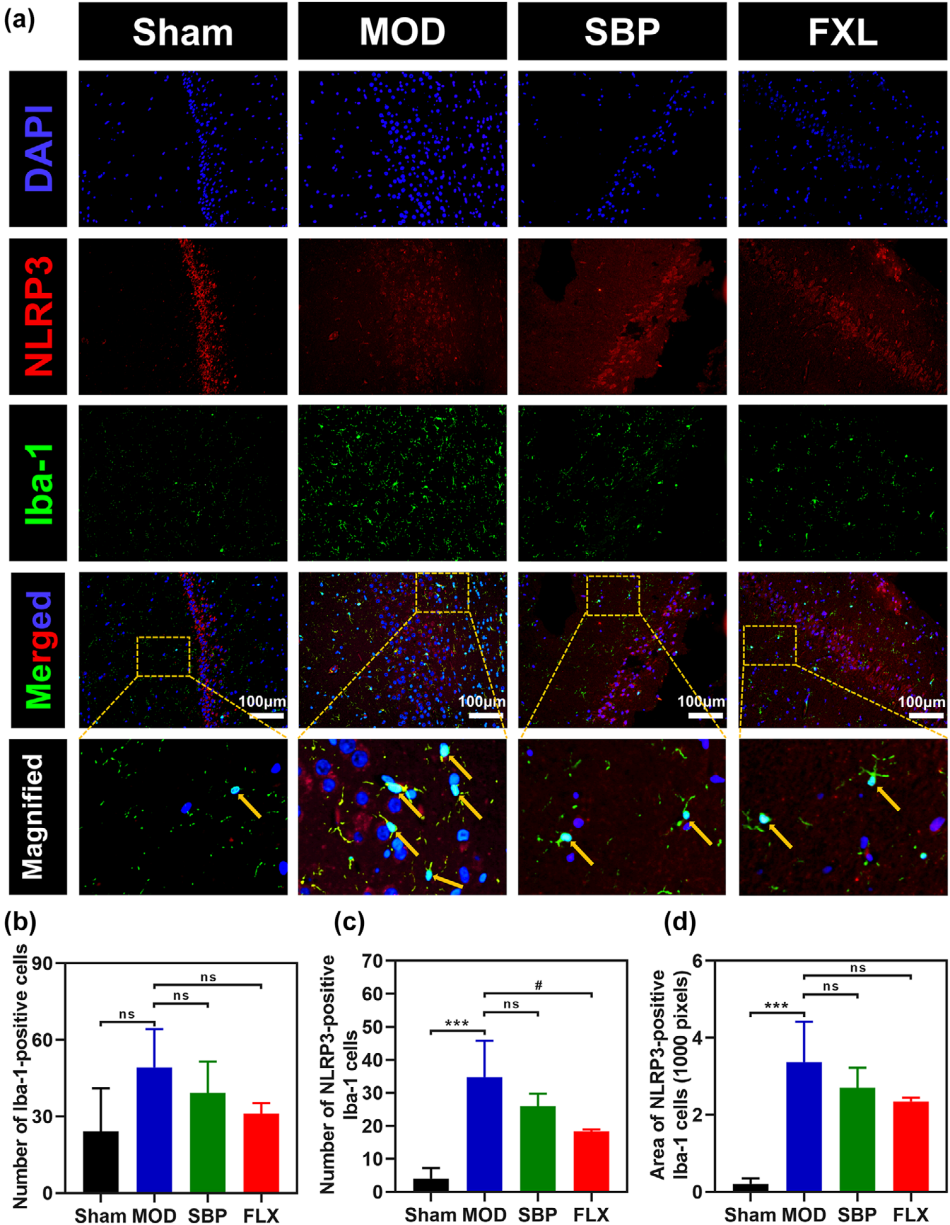
Effect of SBP on microglia and NLRP3 activation in hippocampus. Representative immunofluorescence microphotographs of microglia (Iba‐1, green) and NLRP3 (red)‐positive microglia in hippocampus (a). Nuclei were stained with DAPI (blue). Scale bar = 100 µm. Quantitative analysis of Iba‐1‐positive microglia (b). *n* = 3. Quantitative analysis of NLRP3‐positive Iba‐1‐positive microglia (c, d). *n* = 3. ^*^ *p *<  .05, ^**^ *p *<  .01 vs. Sham group; **^##^** *p *<  .01 vs. MOD; ns, not significant. MOD, model group; SBP, Shexiang Baoxin Pills group; FLX, fluoxetine group.

The microglia‐driven inflammatory response plays a pivotal role in the pathogenesis of neuroinflammation (Kwon et al., 2020). The maturation of IL‐1β is dependent on the activation of NLRP3 inflammasome. To investigate whether microglial and NLRP3 inflammasome activation occurs in rats subjected to CUMS and AMI, as well as the inhibitory effects of SBP on these activations, we evaluated the expression levels of NLRP3, ASC, and Caspase‐1 in the hippocampus using Western blot analysis (Figure 5a, c, d, and e). Our results demonstrated an up‐regulation of NLRP3, ASC, and Caspase‐1 expression in the MOD group; however, treatment with SBP and FLX led to a down‐regulation of varying degrees in their expression levels. Furthermore, we investigated the mRNA level expression of NLRP3 as a crucial component of the NLRP3 inflammasome and provided evidence for its activation along with the inhibitory effect exerted by SBP and FLX on NLRP3 transcription (Figure 5b). Subsequently, microglial activation and NLRP3 expression were assessed using immunofluorescence colocalization analysis. We employed Iba‐1 as a specific marker for microglia and observed that (Figure 6a and b) the rats in the Sham group exhibited a quiescent state of Iba‐1 positive cells, while the MOD group displayed an increased number of Iba‐1 positive cells with enlarged cell bodies. Quantitative analysis revealed a decrease in the number of Iba‐1 positive cells in rats treated with SBP and FLX. Moreover, fluorescence colocalization analysis (Figure 6a, c, and d) demonstrated an elevation in the population of NLRP3‐positive Iba‐1‐positive cells in the MOD group but a significant reduction in both SBP and FLX groups. However, subsequent quantitative analysis indicated no statistically significant difference between the MOD and SBP groups. Collectively, these findings suggest that SBP exerts a certain degree of inhibition on microglial activation and NLRP3 inflammasome activation.

### 3.5 The administration of SBP exhibits a potential inhibitory effect on pyroptosis in hippocampal microglia of rats

**FIGURE 7:**
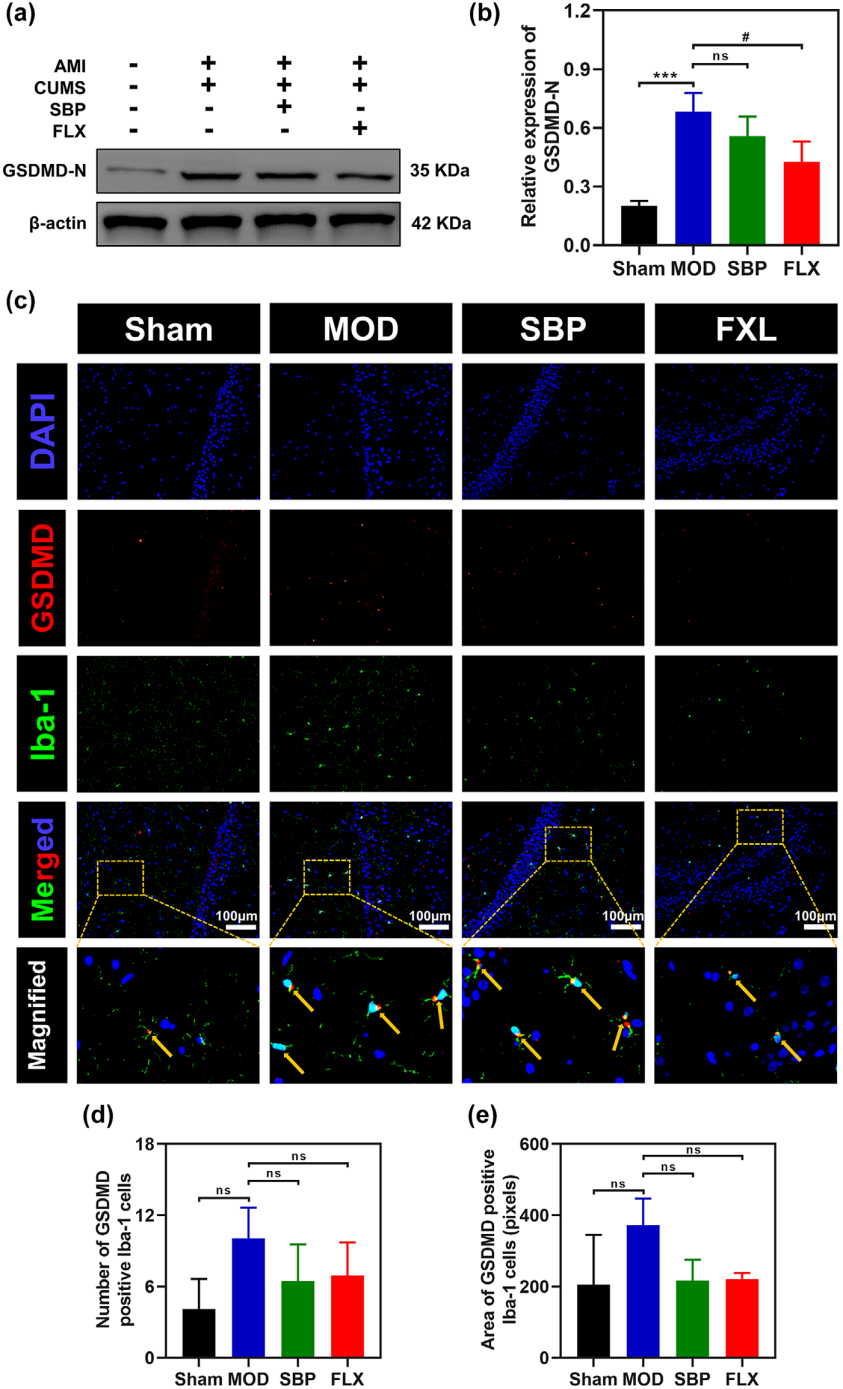
Effect of SBP on pyroptosis of microglial cells in the hippocampus. Representative bands detected by Western blotting for GSDMD‐N (a). Analysis of relative gray values of GSDMD‐N (b). *n* = 3. Representative immunofluorescence microphotographs of microglia (Iba‐1, green) and GSDMD (red)‐positive microglia in hippocampus (c). Nuclei were stained with DAPI (blue). Scale bar = 100 µm. Quantitative analysis of GSDMD‐positive Iba‐1‐positive microglia (d, e). *n* = 3. ***p <  .001 vs. Sham group; ^#^ *p* <  .05 vs. MOD; ns, not significant. MOD, model group; SBP, Shexiang Baoxin Pills group; FLX, fluoxetine group.

The activation of the NLRP3 inflammasome triggers Caspase‐1 maturation, and the active form of Caspase‐1 can initiate cell death through the pyroptosis pathway (S. Li et al., 2021). Based on our experimental findings, we observed a significant upregulation of Caspase‐1 in the MOD group, which was subsequently attenuated following SBP treatment. Subsequently, we assessed the protein expression level of GSDMD, an N‐terminal marker for pyroptosis (Figure 7a and b). The hippocampal expression of GSDMD‐N was observed to increase following CUMS and AMI stimulation, while a decrease was observed after SBP treatment; however, these differences did not reach statistical significance. Immunofluorescence analysis further revealed the colocalization of GSDMD with Iba1‐positive microglia in the hippocampus (Figure 7c), and an elevated number of GSDMD‐positive Iba1‐positive cells were observed in the MOD group, suggesting that CUMS and AMI stimulation may enhance microglial pyroptosis in the hippocampus. The SBP and FLX treatments exhibited a tendency to decrease microglial pyroptosis; however, no statistically significant difference was observed in the quantitative analysis (Figure 7d and e). Overall, these findings suggest that Shexiang Baoxin Pills possess potential inhibitory effects on microglial pyroptosis in the rat hippocampus.

## 4 DISCUSSION

The complexity of TCM's composition poses challenges for identifying specific active pharmaceutical components. However, advancements in technologies such as HPLC/DAD are progressively detecting these active components, significantly aiding in elucidating their specific biological effects. For instance, cinnamaldehyde, a primary active compound in cinnamon, has been observed to exhibit antidepressant‐like behaviors and reduce brain inflammation in CUMS rats (Yao et al., 2015). Moreover, the active compounds Ginsenoside Rg1 and GinsenosideRb3 in ginseng have been reported to display antidepressant‐like effects on CUMS rats. Ginsenoside Rg1 can alleviate inflammatory responses by inhibiting the activation of NLRP3 inflammasome in the hippocampus, thereby achieving antidepressant effects (Bahramsoltani et al., 2015; Cui et al., 2012; He et al., 2023). Ginsenoside Re exhibits anti‐inflammatory effects by inhibiting nitric oxide production and suppressing LPS‐induced NF‐κB signaling in microglia (Wu et al., 2007). Furthermore, it has been ascertained that ginsenoside can cross the blood‐brain barrier (Zhao et al., 2018). The conventional treatment for cardiovascular disease comorbid with depression primarily involves controlling the underlying illness through standardized therapy and administering antidepressants, such as sertraline, deanxit, and fluoxetine. However, these medications not only escalate patients’ financial burdens but also heighten the risk of adverse reactions, and medication adherence cannot be ensured. Employing a holistic approach, Traditional Chinese Medicine (TCM) treats both heart disease and mental health conditions, potentially offering a novel strategy for exploring the prevention and management of cardiovascular diseases.

In our experiments, we initially hypothesized that SBP could mitigate the alterations in depression‐like behaviors by modulating the inflammatory response triggered by AMI and depression. This assumption is grounded on a substantial body of literature indicating that SBP can ameliorate inflammatory responses (Fang et al., 2017; Lin et al., 2023; Tian et al., 2011). Recent studies have demonstrated the crucial role of inflammation in the pathogenesis of postmyocardial infarction depression (Cohen et al., 2015), with inflammation serving as a common pathological basis for both MI and depression (Guo et al., 2023; Kohler et al., 2016; Liberale et al., 2021; Mahtta et al., 2020). Peripheral inflammatory factors reflect the inflammatory status of the central nervous system, capable of penetrating the blood‐brain barrier to directly or indirectly influence central inflammation through active transport and activation of other inflammatory mediators via cerebrovascular endothelial cells (Miller et al., 2009). The elevation in proinflammatory cytokines is associated with reactive oxygen species and reactive nitrogen species production, which subsequently leads to tetrahydrobiopterin oxidation disruption, impairing serotonin (5‐HT), dopamine (DA), and norepinephrine (NE) synthesis while also contributing to impaired motivation and motor activity (Felger, 2018; Frank et al., 2016). Our findings revealed that SBP improved depressive symptoms such as anhedonia and hopelessness while alleviating peripheral and central elevation of IL‐1β and IL‐18 proinflammatory cytokines. The efficacy was comparable to that observed with FLX antidepressant treatment. Similarly, (Zhou et al., 2019b) (reported significant reduction in anhedonic behavior measured using SPT following intragastric administration of SBP at doses of 20.25 or 40.5 mg/kg in CUMS mice; high doses further increased exploration into the central area during OFT.

The NLRP3 inflammasome is a protein complex consisting of intracellular innate immune receptors, Caspase‐1 precursors, and apoptosis‐associated speck‐like protein (ASC). It is predominantly localized in macrophages and microglia. Under conditions of cellular stress, immune cell activation leads to the translocation of ASC from the nucleus to the cytoplasm, where it interacts with the pyrin domain to initiate assembly of the NLRP3 inflammasome. This results in recruitment and self‐cleavage of Pro‐caspase‐1, leading to its activation (Fu et al., 2023; Paik et al., 2021). The activated form of Caspase‐1 functions as a key regulatory protein involved in the mediation of inflammation. It facilitates the cleavage of immature pro‐IL‐1β and pro‐IL‐18, leading to their activation and subsequent release into the extracellular matrix. This process, similar to the NF‐κB pathway, ultimately results in enhanced production of inflammatory factors (Kouba et al., 2022; Zhang et al., 2023). Previous studies have demonstrated that the activation of NLRP3 inflammasome represents a shared underlying mechanism in both coronary heart disease and anxiety and depression (Alcocer‐Gómez et al., 2017). NLRP3 knockout mice did not manifest depressive‐like behaviors, even following prolonged exposure to CUMS (Alcocer‐Gómez et al., 2016). We examined the expression of NLRP3 through Western blot analysis and observed a significant upregulation in the levels of NLRP3, ASC, and Caspase‐1 in the hippocampus of MOD rats. These findings suggest that activation of the NLRP3 inflammasome occurs in individuals with MI accompanied by depression. Previous studies have demonstrated that stress or immune stimulation can induce neuroinflammation, primarily involving microglial activation within the hippocampus (Mahajan et al., 2018). Our immunofluorescence staining revealed that CUMS and AMI induced a significant increase in the number of microglia in the hippocampus, accompanied by cellular hypertrophy, indicative of microglial activation. Notably, NLRP3 bodies were predominantly expressed within the microglial population. Furthermore, treatment with SBP effectively suppressed the activation of NLRP3 inflammasome, consistent with previous findings (Abu‐Elfotuh et al., 2022; Fang et al., 2022b). These findings suggest that the impact of SBP on depression‐like behaviors may mitigate inflammation through a reduction in hippocampal microglial activation and NLRP3 expression.

To the best of our knowledge, pyroptosis represents a form of programmed cell death that is accompanied by an inflammatory response (Mulla et al., 2023). Pyroptosis is characterized by cellular swelling, lysis, and the release of numerous proinflammatory factors, including IL‐1β, IL‐18, ATP, and HMGB1. There are two forms of pyroptosis triggering: GSDMD (gasdermin D)‐dependent activation regulated by caspase‐1/4/5/11 and GSDME‐dependent activation regulated by caspase‐3 (Devant et al., 2023; Hu et al., 2022). NLRP3 inflammasomes assemble and activate Caspase‐1 upon sensing stimuli. This leads to the cleavage of GSDMD into GSDMD‐N and GSDMD‐C fragments. The translocation of GSDMD‐N occurs within ER lobules followed by the extrusion of both plasma membrane lobules to aggregate into oligomers. These events result in cell permeability swelling and spontaneous cell death (Christidi et al., 2021; Xia et al., 2023). Recent studies have demonstrated that CUMS stimulation induces the release of a substantial quantity of damage‐associated molecular patterns (DAMPs), which subsequently upregulate and activate the NLRP3 inflammasome, thereby triggering pyroptosis (Cao et al., 2023; Huang et al., 2023, Z. Zhou et al., 2023). Consistent with our findings, activation of the NLRP3 inflammasome was observed in AMI rats with depression, leading to the cleavage of GSDMD and subsequent release of IL‐18 and IL‐1β, thereby promoting microglial pyroptosis. This effect was partially attenuated by SBP treatment. Hence, we propose that NLRP3 inflammasome‐induced microglial pyroptosis in the hippocampus is a significant contributor to depression‐like behavior. In recent years, pyroptosis has emerged as a prominent research focus due to its involvement in various human diseases. The elucidation of signaling pathway regulation mechanisms has further highlighted the substantial role played by pyroptosis in disease management (Hadian et al., 2023; Jin et al., 2023, Q. Zhou et al., 2023).

Several limitations of this study should be acknowledged. First, the SBP was not categorized into distinct concentration gradients as controls, thus making it difficult to determine whether the antidepressant potency of SBP is comparable to that of conventional drugs. We solely examined SBP as a whole preparation without investigating individual materials and major components that might play significant roles. Second, no control variable analysis was conducted on the molecular mechanism underlying NLRP3 inflammasome activation and its subsequent pyroptosis induction; our study merely identified the existence of this phenomenon. Lastly, there have been no reported clinical studies on the application of SBP in patients with psychiatric disorders. Therefore, further evaluation in a clinical setting is necessary to assess its clinical benefits and risks in preventing depression.

In conclusion, we investigated the impact of SBP treatment on depressive‐like behavior in rats with depression following AMI, potentially achieved through amelioration of neuroinflammation and microglial pyroptosis mediated, at least partially, by the NLRP3/Caspase‐1/GSDMD signaling pathway. First, administration of SBP improved cardiac dysfunction and behavioral impairment while reducing both peripheral and central inflammatory responses after AMI. Second, SBP downregulated the expression of pyroptotic molecules including NLRP3, Caspase‐1, and GSDMD while mitigating microglial activation. This study presents a novel approach for determining the treatment of SBP in MI patients with comorbid depression, which may have significant implications for clinical practice.

## AUTHOR CONTRIBUTIONS

**Yue Wang**: Writing—original draft; software; visualization; data curation. **Yuwen Chen**: Investigation; validation. **Bingqing Li**: Conceptualization; methodology. **Yilu Zhou**: Formal analysis; project administration. **Jing Guan**: Formal analysis; project administration. **Fanke Huang**: Investigation; formal analysis. **Jingjing Wu**: Investigation; methodology. **Yanyan Dong**: Conceptualization; visualization. **Peiyuan Sun**: Conceptualization; investigation; formal analysis. **Xue Tian**: Conceptualization; funding acquisition; writing—review and editing; methodology. **Jindan Cai**: Writing—review and editing; project administration; resources. **Feng Ran**: Project administration; funding acquisition. **Qiuting Dai**: Project administration; resources; investigation. **Jianfeng Lv**: Writing—review and editing; project administration; funding acquisition; resources; supervision; methodology.

## CONFLICT OF INTEREST STATEMENT

The authors declare that they have no competing interests.

### PEER REVIEW

The peer review history for this article is available at https://publons.com/publon/10.1002/brb3.3586.

## Supporting information

Supporting Information

## Data Availability

The data that support the findings of this study are available on request from the corresponding author, JF Lv, upon reasonable request.
