# Supplementary material for: The antidepressant effect of Shexiang Baoxin Pills on myocardial infarction rats with depression may be achieved through the inhibition of the NLRP3 inflammasome pathway
Source: Brain Behav. 2024 Jul 5;14(7):e3586. doi: 10.1002/brb3.3586 (PMC11226411; doi:10.1002/brb3.3586)
Supplement: Supplementary file 1 — Supporting Information [file BRB3-14-e3586-s001.docx]

**Table 1.** **The main chemical components of SBP**

| **Material name** | **Chinese Name** | **Full scientific name** | **Major pharmacologically active components** |
| --- | --- | --- | --- |
| Artificial Mouchus | REN GONG SHE XIANG | The dried preputial secretion of *Moschus berezovskii Flerov,* *Moschus sifanicus Przewalski,* or *Moschus moschiferus Linnaeus* | Muscone, testosterone |
| Calculus Bovis Artifactus | REN GONG NIU HUANG | The dried gall-stone of Bos taurus domesticus Gmelin | Cholic acid, deoxycholic acid, ursodeoxycholic acid, chenodeoxycholic acid, hyodeoxycholic acid, bilirubin, cholesterol |
| Radix Ginseng | REN SHEN | *Panax ginseng* C.A. Mey., root | Ginsenoside Ra1/2, Rb1/2/3, Re, Rc, Rd, Re, Rf, and Rg1/2/3 |
| Venenum Bufonis | CHAN SU | The dried secretion of *Bufo bufo gargarizans Cantor* or *Bufo melanostictus Schneider* | Cinobufagin, resibufogenin, resibufagin, gamabufotalin, bufalin, 1β-hydroxylbufalin, arenobufagin, bufotalin, telocinobufagin, telibufagin |
| Cortex Cinnamomi | ROU GUI | *Cinnamomum cassia* (L.) J. Presl., bark | Cinnamic acid, cinnamaldehyde |
| Styrax | SU HE XIANG | *Liquidambar orientalis* Mill., resin | Benzyl benzoate |
| Borneolum Syntheticum | BING PIAN | *Borneolum Syntheticum* or *Dryobalanops aromatica* C.F. Gaertn, resin | Borneol, isoborneol |

**Supplementary data**

**1 The analytical parameters for the compositional analysis of SBP.**

Liquid chromatography conditions:

Waters C18 column (5μm, 250mm x4.6 mm, Waters, USA); C18 guard column (5μm, 7.5mmx4.6mm, Merck, USA); Mobile phase A (0.5% formic acid): B(acetonitrile), gradient elution, elution procedure: 0-7min, A:80%, B:20%; 7-25min, A:70%, B:30%; 25-35min, A:70%, B:30%; 35-60min, A:65%, B:35%; 60-65min, A:65%, B:35%; 65-75min, A:30%, B:70%; 75-80min, A:0%, B:100%; 80-95min, A:0%, B:100%. The flow rate was 0.8mL/min, the column temperature was 25℃, and the DAD detection wavelengths were 203nm, 280nm and 446nm. (see Supplementary Figure 1.1 for Chromatograms). The injection volume was 10μL. The analysis time was 95min.

Conditions for ESI-MS mass spectrometry:

Electrospray ionization source (ESI), source voltage 3.5kv, drying gas flow rate 10L/min, drying temperature 350℃, spray gas pressure 30psi, target ion molecular weight 600, compound stability 100%, MS break voltage 1.0V, scanning range 100-1500u, MS spectra in positive and negative ion modes were collected separately (see Supplementary Figure 1.2 for mass spectra). CHEMSTATION software was used for data analysis.


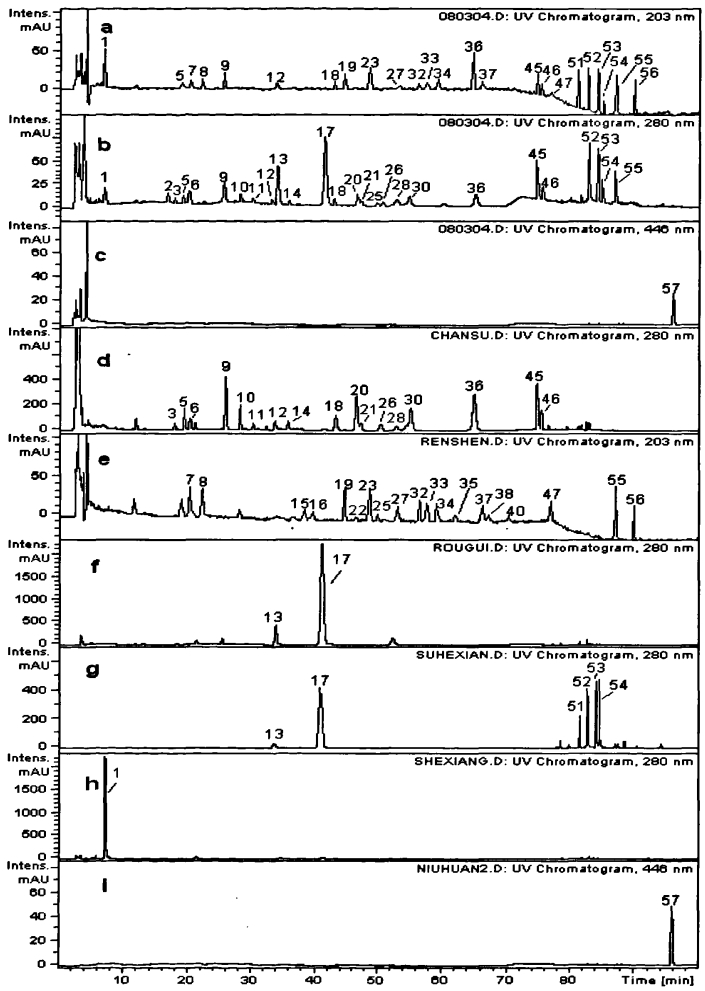


**Supplementary Figure 1.1** (a), (b) and (c) are the DAD chromatograms of the extract of Shexiang Baoxin Pill at 203 nm 280 nm and 446nm. The DAD chromatograms of Venenum Bufonis (d), Radix Ginseng (e), Cortex Cinnamomi (f), Styrax (g), Mouchus (h) and Calculus Bovis Artifactus (i). All chromatographic peaks are the same as those in Table 1.2.


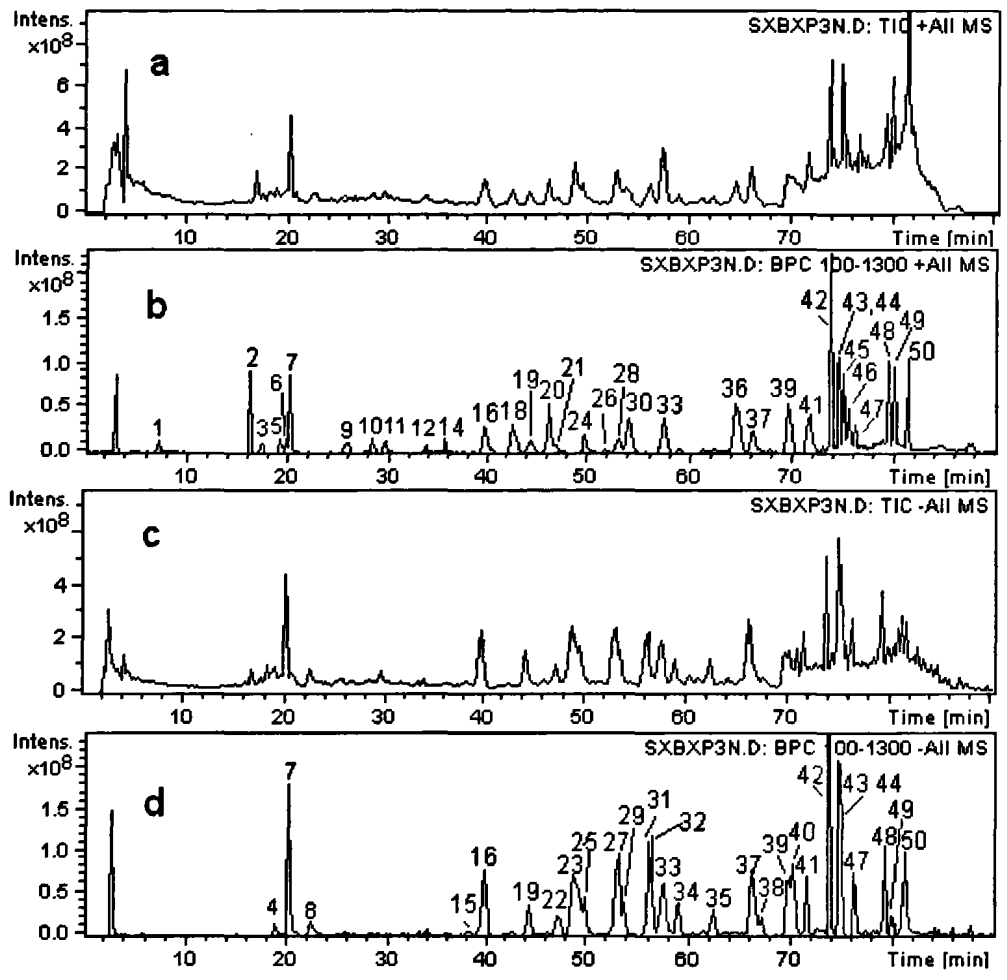


**Supplementary Figure 1.2** TIC diagram of Shexiang Baoxin pill in positive ion mode (a), TIC diagram in negative ion mode (c), BPC diagram in positive ion mode (b), and BPC diagram in negative ion mode (d).

**2 Identification results by HPLC-DAD-MS/MS**

A total of 57 chromatographic peaks were detected by HPLC-DAD-MS, and 47 compounds including ginsenosides, bufondienolactone, cholic acids were finally identified by comparing with the retention time, MS, MS/MS and other data of the standard substance and the literature. Compound spectral data are presented in Table 2.1

| Peak NO. | t_R_  (min) | Compound | λ max  (nm) | MS^+^  (m/z) | MS^-^  (m/z) | Plant material | Fragments ions (m/z) |
| --- | --- | --- | --- | --- | --- | --- | --- |
| 1 | 7.1 | Unknown | 265 | 359; 585 |  | 1 | 342; 278 |
| 2 | 16.7 | Unknown | 250, 300 | 679; 701 |  |  | 661; 435 |
| 3 | 17.4 | Ψ-Bufarenogin* | 250, 300 | 417[M+H] ^+^  855[2M+Na] ^+^ |  | 2 | 399[M+H-H_2_O] ^+^; 363[M+H-3H_2_O] ^+^  335[M+H-3H_2_O-CO] ^+^; 307[M+H-3H_2_O-2CO] ^+^ |
| 4 | 19.0 | Notoginsenoside R1* | -- |  | 932[M-H]^-^  977[M-H+HCOO]^-^ | 3 | 799[M-H-Xyl]^-^; 769[M-H-Glc]^-^;  637[M-H-Glc-Xyl]^-^; 475[Agl]^-^ |
| 5 | 19.2 | Gamabufotalin* | 250, 300 | 403[M+H] ^+^  827[2M+Na] ^+^ |  | 2 | 385[M+H-H_2_O] ^+^; 367[M+H-2H_2_O] ^+^;  349[M+H-3H_2_O] ^+^; 253[M+H-3H_2_O-α-pyr] ^+^ |
| 6 | 19.9 | Bufarenogin | 250, 300 | 417[M+H] ^+^ |  |  | 399[M+H-H_2_O] ^+^; 371[M+H-H_2_O-CO] ^+^; 363[M+H-3H_2_O] ^+^;  353[M300+H-2H_2_O-CO] ^+^; 335[M+H-3H_2_O-CO] ^+^ |
| 7 | 20.3 | Ginsenoside Re* | -- |  | 946[M-H]^-^  991[M-H+HCOO]^-^ | 3 | 799[M-H-Rha]^-^; 783[M-H-Glc]^-^;  637[M-H-GIc-Rha]^-^; 475[Agl]^-^ |
| 8 | 22.2 | Ginsenoside Re* | -- |  | 800[M-H]^-^  845[M-H+HCOO]^-^ | 3 | 637[M-H-Glc]^-^; 475[Agl]^-^ |
| 9 | 25.8 | Arenobufagin* | 260, 305 | 417[M+H] ^+^  855[2M+Na] ^+^ |  | 2 | 399[M+H-H_2_O] ^+^; 371[M+H-H_2_O-CO] ^+^;  353[M+H-2H_2_O-CO] ^+^; 335[M+H-3H_2_O-CO] ^+^ |
| 10 | 28.3 | Hellebrigenin | 250, 300 | 417[M+H] ^+^  855[2M+Na] ^+^ |  | 2 | 363[M+H-H_2_O] +; 353[M+H-H_2_O-CO] ^+^;  335[M+H-3H_2_O-CO] ^+^ |
| 11 | 29.7 | Desacetylcinobufotalin | 250, 300 | 417[M+H] ^+^ |  | 2 | 399[M+H-H_2_O] ^+^; 381[M+H-2H_2_0] ^+^;  363[M+H-3H_2_0] ^+^; 335[M+H-3H_2_0-CO] ^+^ |
| 12 | 33.5 | Bufotalinin | 250, 290 | 415[M+H] ^+^ |  | 2 | 397[M+H-H_2_0] ^+^; 379[M+H-2H_2_0] ^+^; 351[M+H-2H_2_0-CO] ^+^; 361[M+H-3H_2_0] ^+^; 333[M+H-3H_2_0-Co] ^+^ |
| 13 | 33.8 | Cinnamic acid* | 216, 285 |  |  | 4, 5 |  |
| 14 | 35.8 | 19-oxo-Cinobufotalin | 250, 300 | 473[M+H] ^+^ |  | 2 | 395[M+H-H_2_O-HOAC] ^+^; 377[M+H-2H_2_O-HOAC] ^+^;  349[M+H-2H_2_0-HOAC-CO] ^+^ |
| 15 | 38.2 | Gnsenoside Ra3/  Notoginsenoside Fa | -- |  | 1240[M-H]^-^ | 3 | 1107[M-H-Xyl]^-^; 1077[M-H-Glc]^-^; 945[M-H-Xyl-Glc]^-^;  783[M-H-Xyl-2Glc]^-^; 621[M-H-Xyl-3Glc]^-^ |
| 16 | 39.7 | Ginsenoside Rf* | -- |  | 800[M-H]^-^  845[M-H+HCOO]^-^ | 3 | 637[M-H-Glc]^-^; 475[Agl]^-^ |
| 17 | 41.1 | Cinnamaldehyde* | 225, 285 |  |  | 4, 5 |  |
| 18 | 42.7 | 1β-Hydroxylbufalin | 295 | 403[M+H] ^+^  827[2M+Na] ^+^ |  | 2 | 385[M+H-H_2_O] ^+^; 367[M+H-2H_2_O] ^+^; 349[M+H-3H_2_O] ^+^;  339[M+H-2H_2_O-CO] ^+^; 253[M+H-3H_2_O-α-pyr] ^+^ |
| 19 | 44.2 | Notoginsenoside R2 | -- |  | 770[M-H]^-^  815[M-H+HCOO]^-^ | 3 | 637[M-H-Xyl]^-^; 475[Agl]^-^ |
| 20 | 46.3 | Bufotalin | 255, 310 | 445[M+H] ^+^  911[2M+Na] ^+^ |  |  | 349[M+H-2H_2_O-HOAC] ^+^ |
| 21 | 46.8 | Desacetylcinobufagin | 255, 305 | 405[M+H] ^+^ |  | 2 | 383[M+H-H_2_O] ^+^; 365[M+H-2H_2_O] ^+^; 347[M+H-3H_2_O] ^+^;  337[M+H-2H_2_O-CO] ^+^; 319[M+H-3H_2_O-CO] ^+^ |
| 22 | 47.3 | Ginsenoside Ra1/Ra2/Fc | 250, 300 |  | 1210[M-H]^-^ | 3 | 1077[M-H-XyI]^-^; 1047[M-H-Glc]-; 945[M-H-2XyV/(Xyl-Ara)]^-^;  783[M-H-2Xyl-Glc/(Xyl-Ara-Glc)]^-^ |
| 23 | 48.9 | Ginsenoside Rb1* | -- |  | 1108[M-H]^-^  1153[M-H+HCOO]^-^ | 3 | 945[M-H-Glc]^-^; 783[M-H-2Glc]^-^; 621[M-H-3Glc]^-^; 459[Agl]^-^ |
| 24 | 49.2 | Resibufagin* | 250, 300 | 399[M+H] ^+^  421[2M+Na] ^+^ |  | 2 | 381[M+H-H_2_O] ^+^; 353[M+H-H2O-CO] ^+^; 335[M+H-2H_2_O-CO] ^+^;  307[M+H-2H_2_O-2CO] ^+^; 257[M+H-H_2_O-CO-α-pyr] ^+^ |
| 25 | 49.7 | Ginsenoside Rg2 | -- |  | 784[M-H]^-^  829[M-H+HCOO]^-^ | 3 | 637[M-H-Rha]^-^; 621[M-H-Glc]^-^; 475[Agl]^-^ |
| 26 | 51.6 | 19-oxo-Cinobufagin | 250, 300 | 457[M+H] ^+^ |  | 2 | 421[M+H-2H_2_O] ^+^; 379[M+H-H_2_O-HOAC] ^+^;  361[M+H-2H_2_O-HOAC] ^+^; 333[M+H-2H_2_O-HOAC-CO] ^+^ |
| 27 | 53.0 | Ginsenoside Rc* | -- |  | 1078[M-H]^-^ | 3 | 945[M-H-Araf]^-^; 783[M-H-Araf-Glc]^-^;  621[M-H-Araf-2Glc]^-^; 459[Agl]^-^ |
| 28 | 53.2 | Marinobufagin | 250, 300 | 401[M+H] ^+^ |  | 2 | 383[M+H-H_2_O] ^+^; 365[M+H-2H_2_O] ^+^; 347[M+H-3H_2_O] ^+^;  337[M+H-2H_2_O-CO] ^+^; 319[M+H-3H_2_O-CO] ^+^; 269[M+H-2H_2_O-α-pyr] ^+^ |
| 29 | 53.7 | Ginsenoside Ra1/Ra2/Fc | -- |  | 1210[M-H]^-^ | 3 | 1077[M-H-Xyl]^-^; 1047[M-H-Glc]^-^; 945[M-H-2Xyl/(Xyl-Ara)]^-^  783[M-H-2Xyl-Glc/(Xyl-Ara-Glc)]^-^ |
| 30 | 54.2 | Cinobufotalin | 250, 300 | 459[M+H] ^+^  939[2M+Na] ^+^ |  | 2 | 417[M+H-CH_2_CO] ^+^; 381[M+H-2H_2_O-CH_2_CO] ^+^;  363[M+H-3H_2_O-CH_2_CO] ^+^ |
| 31 | 55.7 | Ginsenoside Ra1/Ra2/Fc | -- |  | 1210[M-H]^-^ | 3 | 1077[M-H-Xyl]^-^; 1047[M-H-Glc]^-^; 945[M-H-2XyV/(Xyl-Ara)]^-^;  783[M-H-2Xyl-Glc/(Xyl-Ara-Glc)]^-^ |
| 32 | 56.2 | Ginsenoside Ro | -- |  | 955[M-H]^-^ | 3 | 793[M-H-Glc]^-^; 613[M-H-2Glc-H_2_O]^-^; 455[Agl]^-^ |
| 33 | 57.6 | Ginsenoside Rb2 | -- |  | 1078[M-H]^-^  1114[M-H+2 H_2_O]^-^ | 3 | 945[M-H-Arap]^-^; 783[M-H-Arap-Glc]^-^;  621[M-H-Arap-2Glc]^-^; 459[Agl]^-^ |
| 34 | 59.0 | Ginsenoside Rb3* | -- |  | 1078[M-H]^-^  1114[M-H+2 H_2_O]^-^ | 3 | 945[M-H-Xyl]^-^; 783[M-H-Xyl-Glc]^-^;  621[M-H-Xyl-2Glc]^-^; 459[Agl]^-^ |
| 35 | 62.2 | Quinquenoside R1 | -- |  | 1150[M-H]^-^ | 3 | 1107[M-CH_3_CO]^-^; 987[M-H-Glc]^-^; 945[M-GlcAc]^-^;  927[M-H-GlcAc-H_2_O]^-^; 783[M-H-GlcAc-Glc]^-^ |
| 36 | 64.4 | Bufalin* | 255, 300 | 387[M+H] ^+^  795[2M+Na] ^+^ |  | 2 | 369[M+H-H_2_O] ^+^; 351[M+H-2H_2_O] ^+^; 333[M+H-3H_2_O] ^+^;  305[M+H-3H_2_O-CO] ^+^; 255[M+H-2H_2_O-α-pyr] ^+^ |
| 37 | 66.0 | Ginsenoside Rd* | -- |  | 946[M-H]^-^  991[M-H+HCOO]^-^ | 3 | 783[M-H-Glc]^-^; 621[M-H-2Glc]^-^; 459[Agl]^-^ |
| 38 | 66.8 | Ginsenoside Rs1/Rs2 | -- |  | 1120[M-H]^-^ | 3 | 1077[M-H-CH_2_CO]^-^; 1059[M-H-H_2_O-CH_2_CO]^-^;  945[M-H-Arap(Araf)-CH_2_CO]^-^;  927[M-H-Arap(Araf)-H_2_O-CH_2_CO]^-^; 459[Agl]^-^ |
| 39 | 69.5 | Unknown | -- | 462; 480 | 514; 1030 |  |  |
| 40 | 70.5 | Ginsenoside Rs1/Rs2 | -- |  | 1120[M-H]^-^ | 3 | 1077[M-H-CH_2_CO]^-^; 1059[M-H-H_2_O-CH_2_CO]^-^;  945[M-H-Arap(Araf)-CH_2_CO]^-^; 927[M-H-Arap(Araf)-H_2_O-CH_2_CO]^-^; 459[Agl]^-^ |
| 41 | 71.5 | Unknown | -- | 355; 373 | 453; 815 |  |  |
| 42 | 73.6 | Cholic acid* | -- |  | 407[M-H]^-^  815[2M-H]^-^ | 6 | 371[M-H-2H_2_O]^-^; 353[M-H-3H_2_O]^-^; 344[M-H-H_2_O-HCOO]^-^;  326[M-H-2H_2_O-HCOO]^-^ |
| 43 | 74.5 | Ursodeoxycholic acid* | -- |  | 391[M-H]^-^  783[2M-H]^-^ | 6 | 373[M-H-H_2_O]^-^; 355[M-H-2H_2_O]^-^; 328[M-H-H_2_O-HCOO]^-^ |
| 44 | 74.5 | Hyodeoxycholic acid* | -- |  | 391[M-H]^-^  783[2M-H]^-^ | 6 | 373[M-H-H_2_O]^-^; 355[M-H-2H_2_O]^-^; 328[M-H-H_2_O-HCOO]^-^ |
| 45 | 74.8 | Cinobufagin* | 250, 300 | 443[M+H] ^+^ |  | 2 | 401[M+H-CH_2_CO] ^+^; 383[M+H-HOAC] ^+^;  365[M+H-HOAC-H_2_O] ^+^; 347[M+H-HOAC-2H_2_O] ^+^ |
| 46 | 75.1 | Resibufogenin* | 250, 300 | 385[M+H] ^+^ |  | 2 | 367[M+H-H_2_O] ^+^; 349[M+H-2H_2_O] ^+^; 321[M+H-2H_2_O-CO] ^+^;  253[M+H-2H_2_O-α-pyr] ^+^ |
| 47 | 76.7 | Ginsenoside Rg3* | -- |  | 784[M-H]^-^  829[M-H+HCOO]^-^ | 3 | 621[M-H-Glc]^-^; 459[Agl]^-^ |
| 48 | 79.2 | Chenodeoxycholic acid* | -- |  | 391[M-H]^-^  783[2M-H]^-^ | 6 | 373[M-H-H_2_O]^-^; 355[M-H-2H_2_O]^-^; 328[M-H-H_2_O-HCOO]^-^ |
| 49 | 79.8 | Deoxycholic acid | -- |  | 391[M-H]^-^  783[2M-H]^-^ | 6 | 373[M-H-H_2_O]^-^; 355[M-H-2H_2_O]^-^; 328[M-H-H_2_O-HCOO]^-^ |
| 50 | 81.2 | Unknown | -- |  |  | 1 |  |
| 51 | 81.6 | Benzyl benzoate* | 240, 270 |  |  | 5 |  |
| 52 | 82.7 | Unknown | 280 |  |  | 5 |  |
| 53 | 84.0 | Unknown | 280 |  |  | 5 |  |
| 54 | 85.5 | Unknown | 225 |  |  | 5 |  |
| 55 | 87.0 | Unknown | -- |  |  | 3 |  |
| 56 | 90.3 | Unknown | -- |  |  | 3 |  |
| 57 | 95.9 | Unknown | 446 |  |  | 6 |  |

**Supplementary Table 2.1** The spectral data of compounds. (a) * indicates identification by comparison of standards. (b) α-pyr represents the α-pyran ring. (c) Agl stands for aglycone. (d) 1：Mouchus；2：Venenum Bufonis；3：Radix Ginseng；4：Cortex Cinnamomi；5：Styrax；6：Calculus Bovis
